# Protective effects of mitochondrial fission inhibition on ox-LDL induced VSMC foaming via metabolic reprogramming

**DOI:** 10.3389/fphar.2022.970151

**Published:** 2022-09-02

**Authors:** Yijin Fang, Yu Zhu, Yue Wu, Liangming Liu, Huadong Wang

**Affiliations:** ^1^ Department of Pathophysiology, Key Laboratory of State Administration of Traditional Chinese Medicine of the People’s Republic of China, School of Medicine, Jinan University, Guangzhou, China; ^2^ State Key Laboratory of Trauma, Burns and Combined Injury, Shock and Transfusion Department of Research Institute of Surgery, Daping Hospital, Army Medical University, Chongqing, China

**Keywords:** foaming, mitochondrial fission, metabolic reprogramming, atherosclerosis, lipid deposition

## Abstract

Atherosclerosis (AS) is one of the most common diseases in middle-age and elderly population. Lipid metabolism disorder induced foaming of vascular smooth muscle cell (VSMC) is an important pathological process of AS. Mitochondria plays an important role in lipids metabolism. While it is not known whether regulating mitochondrial function can protect ox-LDL induced VSMC foaming via metabolic reprogramming. With ox-LDL induced mouse model of VSMC injury, the injury effect of ox-LDL and the protective effect of mdivi-1, the mitochondrial fission inhibitor on mitochondrial morphology and function of VSMC, and the formation of lipid droplet were observed. With metabonomics and proteomics techniques, the main lipid metabolites and regulation proteins were identified. The results showed that Ox-LDL induced a significant mitochondrial fission and fragmentation of VSMC, and mitochondrial function disorder along with lipid deposition and foaming. Mdivi-1 significantly antagonized the damage effect of ox-LDL on mitochondrial morphology and function of VSMC, and blocked the lipid deposition. Metabonomics analysis found 848 different metabolites between ox-LDL and mdivi-1 treatment group, in which the lipid metabolites were the main, and heptadecanoic acid, palmitoleic acid and myristic acid were the critical metabolites changed most. Proteomics results showed that there were 125 differential expressed proteins between ox-LDL and mdivi-1 treatment, acetyl -CoA carboxylase1 and fatty acid synthase were the main differential expressed proteins. This study suggest that Mitochondrial fission plays an important role in VSMC lipid deposition and foaming. Inhibition of mitochondrial fission may effectively fight against ox-LDL induced lipid deposition and foaming of VSMC via improving mitochondrial function and metabolic reprogramming. This finding provides a new insight for prevention and treatment of AS.

## Introduction

The incidence and mortality of cardiovascular diseases are still increasing all over the world. Atherosclerosis (AS) is one of the most common diseases in the middle-aged and elderly population, and it is also the key point of many diseases, such as vascular lipid deposition, stenosis, and cerebrovascular blockage (Libby et al., 2019). Among the many factors leading to AS, hyperlipidemia, was considered to be a vital inducement. The incidence and development of AS involves multiple mechanisms, including endothelial dysfunction, inflammation, oxidative stress and so on (Katakami, 2018). The present treatment measures for AS are mainly focused on lowering blood lipid, anticoagulation, and thrombolysis, but the treatment effect is not ideal. Therefore, it is of importance to explore the new targets for the treatment of AS.

The theory of “inflammation-injury-response” for the pathogenesis of AS is widely recognized, which complements the “lipid permeation theory” and explains the whole process of the occurrence and development of AS (Wolf and Ley, 2019). The formation of foaming cells is a hallmark of AS. There are many sources of foaming cells involving in AS, such as mononuclear macrophages, vascular smooth muscle cells (VSMCs) and so on, which can be transformed into foaming cells and participate in the occurrence of AS (Maguire et al., 2019). In the middle and late stages of AS, the foaming cells mostly come from VSMCs, while at the early stage of AS, the foaming cells mainly come from mononuclear macrophages (Luo et al., 2017). Present studies indicate that the formation of foaming cell is mainly induced by intracellular lipid accumulation. Previous studies demonstrated that lipid accumulation in VSMC following AS was related to the increase of lipid level in circulation, which entered into the cell via LDL-R (low density lipoprotein receptor) or scavenger receptor (SR), CD36, CD68 and so on (Pryma et al., 2019). However, basic research demonstrated that the synthesis of lipids via acetyl CoA from mitochondria was another pathway taking part in the balance of lipids level in cells. Present treatments for AS are mainly focused on reducing exogenous lipids absorption, there are no effective methods to regulate the endogenous lipids production for treatment of AS.

Mitochondria are the important organelle and the energy supplier of the cells. Glucose, lipids, and amino acids can be metabolized in mitochondria via forming acetyl CoA and participate in tricarboxylic acid cycle (TCA) to provide energy for cells. Excessive acetyl CoA could enter the cytoplasm through the cycle of malic acid, and stirs up the production of lipids, resulting in lipids accumulation and cell foaming (Sukhorukov et al., 2020). Mitochondrial function may directly modulate the metabolic level of mitochondria. Basic studies showed that mitochondrial morphology and structure change played a decisive role in mitochondrial function (Faitg et al., 2020). Many stimulations, such as hypoxia, lipopolysaccharide, starvation could disturb mitochondrial morphology, resulting in the over fission of mitochondria, and finally affected the mitochondrial function including metabolic function (Benador et al., 2019). However, whether mitochondrial morphology is disturbed during AS development, and whether modulating mitochondrial morphology will play an inhibitory role in the formation of VSMC foaming are unknown.

So, ox-LDL was used to stimulate mouse VSMC to observe the effect of mitochondrial fission inhibitor mdivi-1 on lipid deposition. And the mechanisms were explored by metabolomics and proteomics. The results of present study will provide a new direction for the treatment of AS.

## Materials and methods

### Reagent

Acetyl -CoA carboxylase 1 and Fatty acid synthase antibody were purchased from Bostor biological technology. MitoTracker™ was purchased from invitrogen, oxidized low density lipoprotein (ox LDL) was from Yiyuan Biotechnologies, 2′,7′-Dichlorodihydrofluorescein diacetate (DCFH-DA) were purchased from Abcam (Cambridge, MA, United States). Oil Red O were purchased from Jiancheng Co, Nanjing, China.

### Lipid deposition measurement

The lipid deposition of VSMC (from American Type Culture Collection) was measured by Oil Red O staining. According to operating manual, ox-LDL (50 μg/ml) (Wu et al., 2020). Treated VSMCs were washed three times with PBS solution, and then the Oil Red O and 4′,6-diamidino-2-phenylindole (DAPI) were added and incubated for 15 and 3 min, respectively, and then used for observation.

### Mitochondrial morphology observation

2×10^4^ VSMCs were inoculated in a confocal culture dish and when grew to 60%–70% fusion, they were used for experiments. After treatment with ox-LDL (50 μg/ml) and mdivi-1 (20 mg/ml), the cells medium was completely removed, and washed 3 times with sterile PBS. MitoTracker™ Deep Red (100 nmol/L) was added to cell and incubated in 5% CO_2_ at 37°C for 30 min, the cells were observed and imaged by laser confocal microscope (Leica TCS SP5, Germany). The red fluorescence excitation wave is 633 nm and the visible range is 655–670 nm. ImageJ software was used to quantitatively analyze the length and number of mitochondrial branches, and manual analysis was carried out with reference to MINA plug-in. The detail methods were as: the mitochondrial image obtained by the confocal system were imported into ImageJ. After the image was modified to 8bit, the threshold was adjusted to two values. Skeletonize was selected in the process, and then used to analyze in the analysis tool. The number and length data of mitochondrial branches, the mitochondrial skeleton were obtained.

### Analysis of mitochondrial fission and fusion

2×10^4^ VSMCs were seeded into the confocal chamber and incubated with Mito-tracker (1:10, 000) at 37°C for 30 min. The Time-Lapse file by confocal microscope was processed by LasX software and cut by Lightroom software, and then exported to GIF format file by FIJI software. Researchers manually calculated the mitochondrial dynamic events in the cell for at least 5 min. Mitochondrial fission was defined as the complete fission of a mitochondrial into two independent daughter mitochondria in at least 30 s. When two mitochondria were in stable contact for more than 30 s and moved together, it was calculated as a fusion.

### Mitochondrial oxygen consumption rate

The oxygen consumption rate (OCR) was measured by Seahorse (Agilent Cell Analysis technology, Unites States) using a 96-well XFe plates (Zhao et al., 2021). VSMCs were seeded at a density of 1×10^4^ per well. The basic assay medium contains 2.5 μM glucose and 2 mM glutamine. After cultured for 1h, injections of 2 μM oligomycin, 1 μM FCCP, and 0.5 μM rotenone/antimycin A were performed sequentially. The OCR was measured by extracellular flux analyzer under the mitochondrial stress test condition.

### Metabonomics and proteomics analysis

To explore the mechanism that mdivi-1 inhibit VSMC formation, metabonomics and proteomics were further performed. Through the integration and analysis of proteomics and metabolomics data, we will grasp the basic state and comprehensively discover potential mechanisms. Ultra-performance liquid chromatography tandem mass spectrometry (LC-MS/MS) developed by BioTree (Shanghai, China) was used to analyze the cells. Cells were extracted with extract solution (acetonitrile: methanol: water = 2: 2: 1, with isotopically-labelled internal standard mixture) and subjected to LC-MS/MS analysis. The metabolites and protein standard solutions were prepared and analyzed by LC-MS/MS to obtain the calibration curve respectively. The metabolites and protein were quantified according to the calibration curves. The production of metabolites and protein were reported according to the relative value (Zhang et al., 2020).

### Bioinformatics analysis

Principal component analysis (PCA) was performed to examine intrinsic clusters of metabolomics data. A 95% confidence interval (CI) was used as the threshold to identify potential outliers in all samples. In addition, heat maps were displayed by the R software package p heat map to visualize the metabolite difference with in the data set. Volcano and violin plots were made using GraphPad Prism V.8.0.0 Software, which directly showed the upregulated and down regulated metabolites. MetaboAnalyst 2 was applied for pathway enrichment analysis.

### Statistical analysis

SPSS 20.0 (SPSS Inc., Chicago, IL, Unites States) was used for statistical analysis. The data were represented as means ± SD. An independent sample t-test was used to analyze the difference between the two groups. One-way analysis of variance (ANOVA) and post hoc test (S-N-K/LSD) were used for more than two groups. *p* < 0.05 was considered statistically significant.

## Results

### Effects of mitochondrial fission inhibition on mitochondrial structure and function of VSMC following ox-LDL stimulation

**FIGURE 1:**
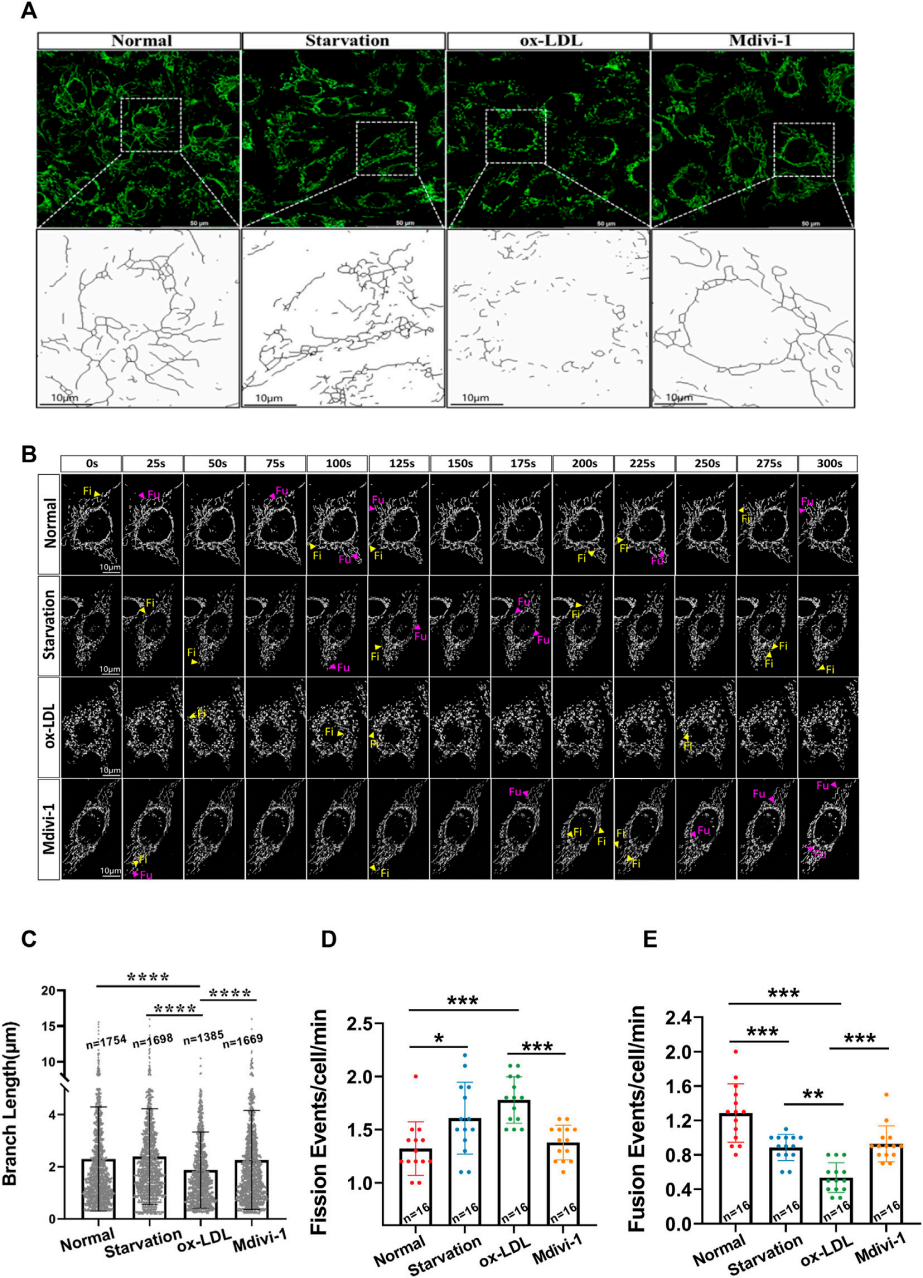
Effects of Mdivi-1 on ox-LDL-induced mitochondrial morphology. **(A)**: The observation of mitochondrial structure, VSMC after treatment was incubation with MitoTracker™ Deep Red (100 nmol/L) for 30 min, the cells were observed and imaged by laser confocal microscope. **(B)**: The Branch Length of mitochondria, the mitochondrial image obtained by the confocal system were imported into ImageJ. Skeletonize was selected in the process, and then used to analyze in the analysis tool. The number and length data of mitochondrial branches, the mitochondrial skeleton was obtained. **(C)**: observation of mitochondrial Time-Lapse, the Las X software and Lightroom software were used to analyze the Time-Lapse file. Researchers manually calculated the mitochondrial dynamic events in the cell for at least 5 min **(D,E)**: mitochondrial fission and fusion frequency, that mitochondria were completely divided into two independent daughter in at least 30 s was defined as mitochondrial fission, at same time, that two mitochondria were in stable contact for more than 30 s and moved together was calculated as a fusion. Fi: fission; Fu: fusion; n: statistical number of mitochondria. *p < 0.05, **p < 0.01, ***p < 0.001.

Mitochondrial structure determines mitochondrial function. Mitochondrial fission and fusion play a vital role in the mitochondrial structure regulation. Mitochondrial excessive fission may lead to mitochondrial dysfunction. Whether hyper-lipid stimulation can induce mitochondrial over-fission, and inhibition of mitochondrial fission is beneficial to hyper-lipid induced lipid accumulation are not clear. As a classical mitochondrial fission inhibitor, Mdivi-1 20 mg/ml was used to explore the effects of inhibition of mitochondrial fission on the VSMC lipid accumulation after ox-LDL treatment. VSMCs were incubated with Ox-LDL 50 μg/ml for 48 h, and followed by Mdivi-1 20 mg/ml for 24 h, the mitochondrial structure and function were observed. The results showed that Ox-LDL stimulation led to the mitochondrial over-fission, it was presented the decrease of branch length of mitochondria, the branch length was decreased from 2.36 ± 1.96 (μm) in normal group to 1.72 ± 1.23 (μm) in Ox-LDL group; At the same time, the frequencies of mitochondrial fission and fusion were disordered significantly. At same time the frequencies of mitochondrial fission were increased from 1.38 ± 0.68 (events/cell/min) to 1.78 ± 0.48 (events/cell/min) following Ox-LDL stimulation, and frequencies of mitochondrial fusion were decreased from 1.31 ± 0.41 (events/cell/min) to 0.58 ± 0.23 (events/cell/min), respectively. Mdivi-1 20 mg/ml treatment significantly antagonized ox-LDL-induced mitochondrial excessive fission, the mitochondrial branch length was increased to 2.34 ± 1.88 (μm), and the frequencies of mitochondrial fission were 1.41 ± 0.36 (events/cell/min), while the frequencies of mitochondrial fusion were 0.98 ± 0.37 (events/cell/min) (Figure 1).

**FIGURE 2:**
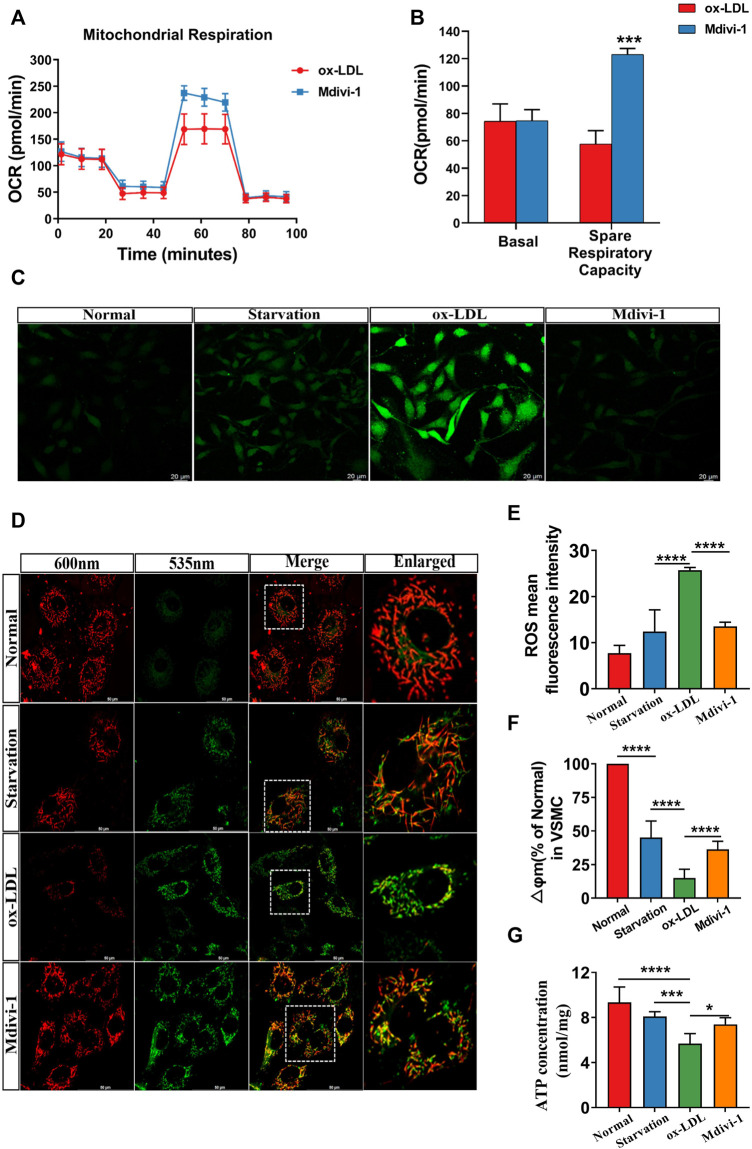
Effects of Mdivi-1 on mitochondrial function. **(A,B)**: The changes of OCR, VSMCs were seeded in a 96-well XFe plates, after cultured with 2.5 μM glucose and 2 mM glutamine for 1h, 2 μM oligomycin, 1 μM FCCP, and 0.5 μM rotenone/antimycin A were performed sequentially. The OCR was measured by extracellular flux analyzer under the mitochondrial stress test condition. **(C,E)**: The changes of ROS. **(D,F)**: The changes of mitochondrial membrane potential. **(G)**: The changes of ATP. OCR: oxygen consumption rate. *p < 0.05, **p < 0.01, ***p < 0.001.

As the main parameters of mitochondrial function, mitochondrial membrane potential (∆Ψm), ROS level and OCR were measured. The results showed that as compared with the normal group, ox-LDL stimulation led to the decrease of ∆Ψm, OCR and ATP level and the increase of ROS. Mdivi-1 administration antagonized ox-LDL induced the disorder of mitochondrial function, the ∆Ψm, OCR and ATP levels were significantly improved, while the ROS was significantly alleviate (*p* < 0.01) as compared with the ox-LDL group (Figure 2).

### Protective effects of mitochondrial fission inhibition on ox-LDL inducing lipid deposition in VSMC

**FIGURE 3:**
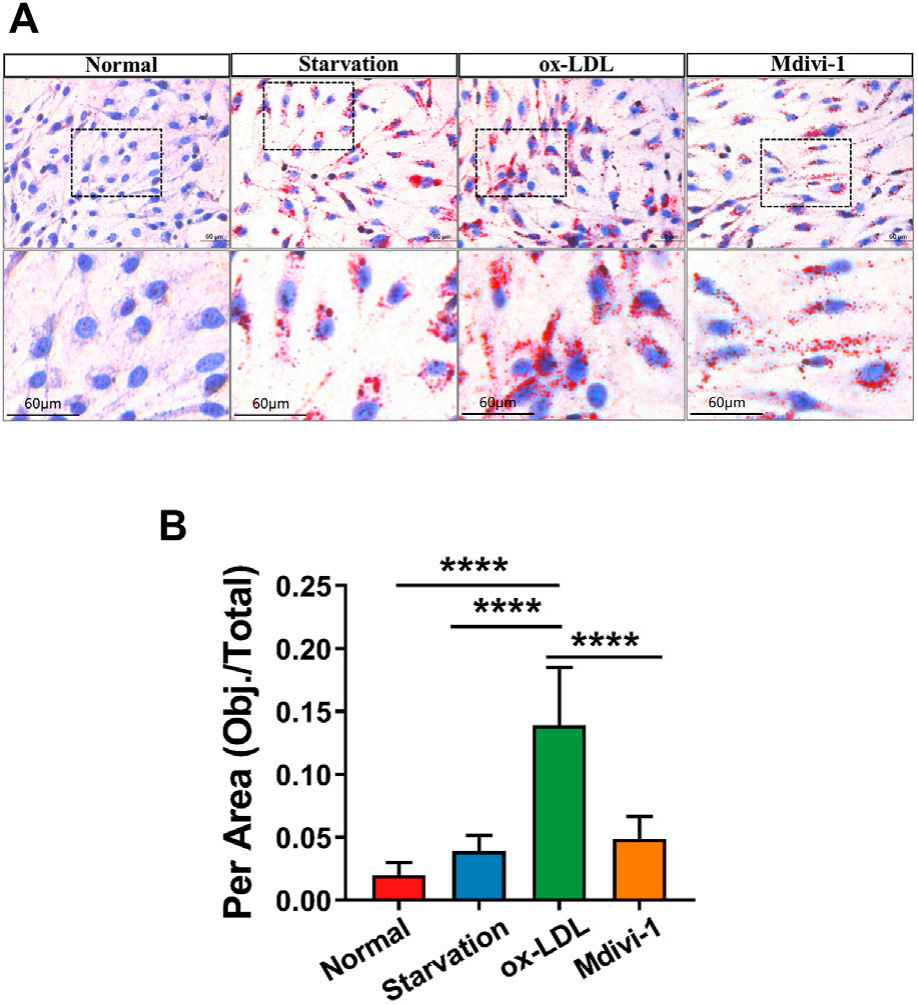
Effects of Mdivi-1 on lipid drop in VSMC. **(A)**: The changes of Oil red O staining, the treated VSMCs were washed with PBS solution, and then the Oil Red O and 4′,6-diamidino-2-phenylindole (DAPI) were added and incubated for 15 and 3 min, respectively. **(B)** Statistical value of oil red staining. *p < 0.05, **p < 0.01, ***p < 0.001.

In order to investigate the effect of inhibition of mitochondrial fission on lipid deposition in VSMC. The effect of Mdivi-1 on lipid deposition were observed. . The results showed that ox-LDL incubation significantly increased the lipid level in VSMC with Oil Red O staining, it was showed that red marker lipid was significantly increased with Oil Red O staining. Mdivi-1 20 mg/ml treatment significantly antagonized ox-LDL induced lipid accumulation in VSMC (Figure 3). The results indicated that mitochondrial fission inhibitor antagonized ox-LDL-induced lipid deposition.

### Effect of Mdivi-1 on ox-LDL induced change of metabonomics in VSMC

**FIGURE 4:**
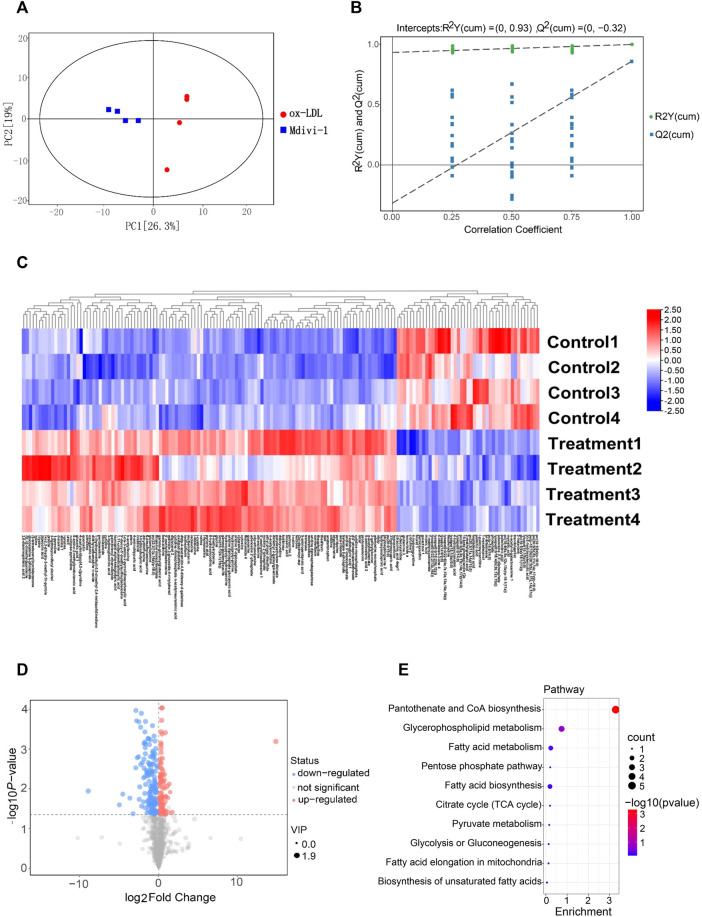
The landscape of metabolism profile of heart following inflammation in rats. **(A)**: Principal component analysis (PCA) scores plot for metabolomics analysis in Mdivi-1 and ox-LDL group. A 95% confidence interval (CI) was used as the threshold to identify potential outliers in all samples. Orange represented mdivi-1 group, and blue represent ox-LDL group. **(B)**: Permutation test of OPLS-DA model for Mdivi-1 vs. ox-LDL group. **(C)**: Heat map analyzed showing the significantly changed metabolites in Mdivi-1 vs. ox-LDL group, the R software package p heat map to visualize the metabolite difference with in the data set. **(D)**: Volcano plot, Volcano plots were made using GraphPad Prism V.8.0.0 Software. **(E)**: metabolic pathway enrichment analysis identified in Mdivi-1 vs. ox-LDL group. The different color depth of circles represents the *p* value of pathway enrichment analysis.

To explore whether Mdivi-1 inhibit lipid deposition in VSMCs after ox-LDL treatment through metabolic reprogramming, the metabonomics profiling of VSMCs following ox-LDL and Mdivi-1 treatments were examined. The samples were completely different by Principal component analysis (PCA) plots (all samples were within the confidence interval) (Figure 4A). The stability and reliability of the model were confirmed by results of OPLS-DA (Figure 4B). The volcano plot showed that there were 848 different metabolites between the ox-LDL incubation and Mdivi-1 treatment (Figure 4D) (Metabolites were selected by VIP value >1 and *p* value <0.05). Hierarchical clustering heatmap results showed that ox-LDL incubation significantly activated the lipid metabolic pathways such as pantothenate and CoA biosynthesis, glycerophospholipid metabolism, fatty acid metabolism, and fatty acid biosynthesis, and so on. And several lipid metabolites were increased (Figures 4C,E).

**FIGURE 5:**
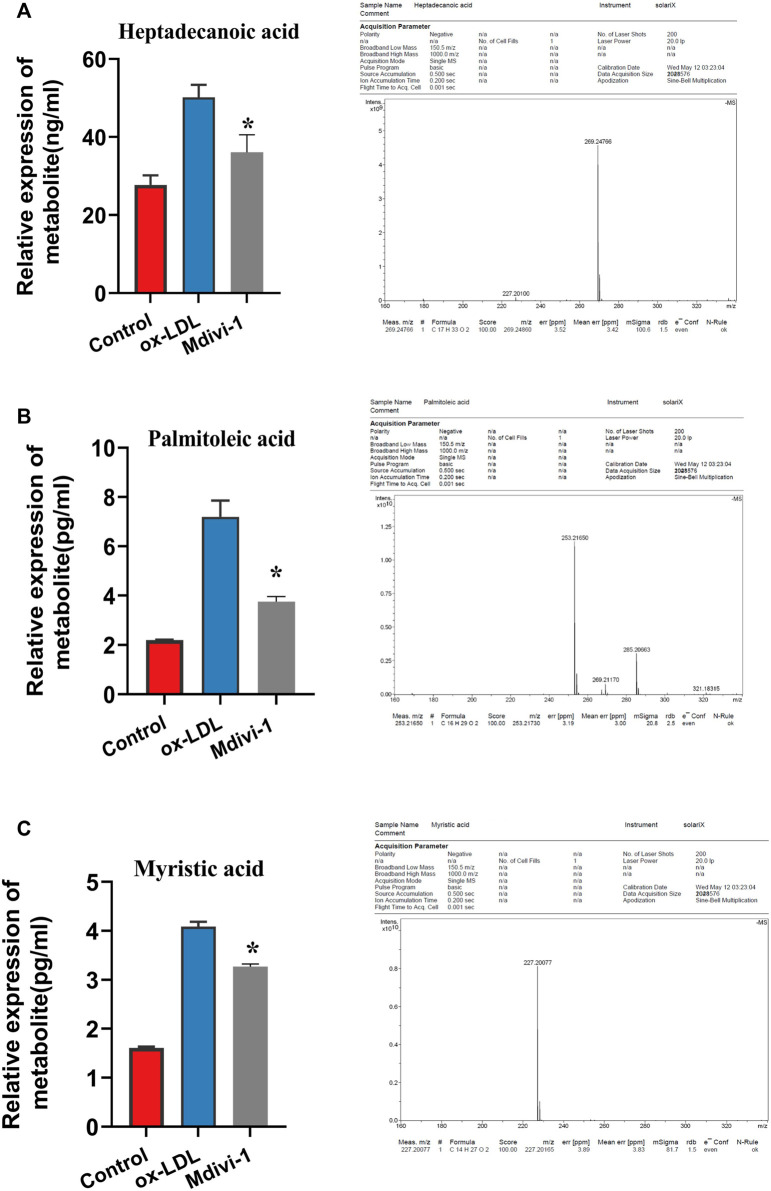
Verification of heptadecanoic acid, palmitoleic acid, myristic acid after Mdivi-1 treatment in VSMC, the treated VSMC were centrifugated to remove the culture medium and then was sample loaded to measure by MS. **(A)**: heptadecanoic acid. **(B)**: palmitoleic acid. **(C)**: myristic acid. *p < 0.05.

In order to further verify the effect of ox-LDL and Mdivi-1 on lipid metabolism of VSMC, the metabolites of VSMCs after ox-LDL and Mdivi-1 treatment were observed by mass spectrum. The results showed that Mdivi-1 significantly inhibited ox-LDL induced increase of fatty acid metabolites including heptadecanoic acid, palmitoleic acid and myristic acid (Figure 5). The results indicated that Mdivi-1 antagonized the ox-LDL-induced VSMC lipid drop formation via abolishing mitochondrial over-fission.

### Effects of Mdivi-1on the proteomics of ox-LDL treated VSMC

**FIGURE 6:**
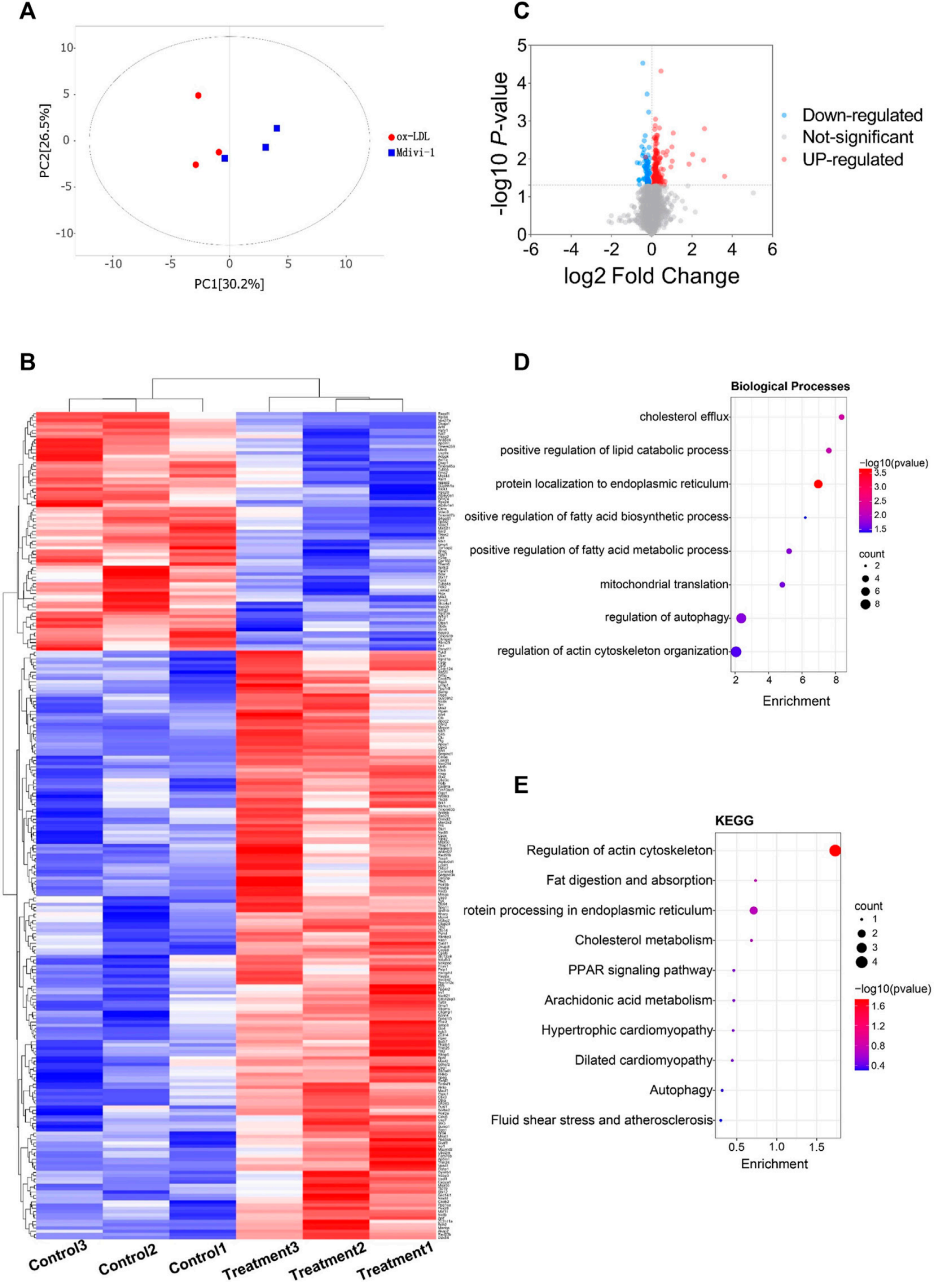
The landscape of protein profile of heart following inflammation in rats. **(A)**: Permutation test of OPLS-DA model for Mdivi-1 vs. ox-LDL group. **(B)**: Heat map analyzed by TBtools showing the significantly changed metabolites in Mdivi-1 vs. ox-LDL group. **(C)**: Volcano plot. **(D,E)**: protein pathway enrichment analysis identified in Mdivi-1 vs. ox-LDL group. The different color depth of circles represents the *p* value of pathway enrichment analysis.

**FIGURE 7:**
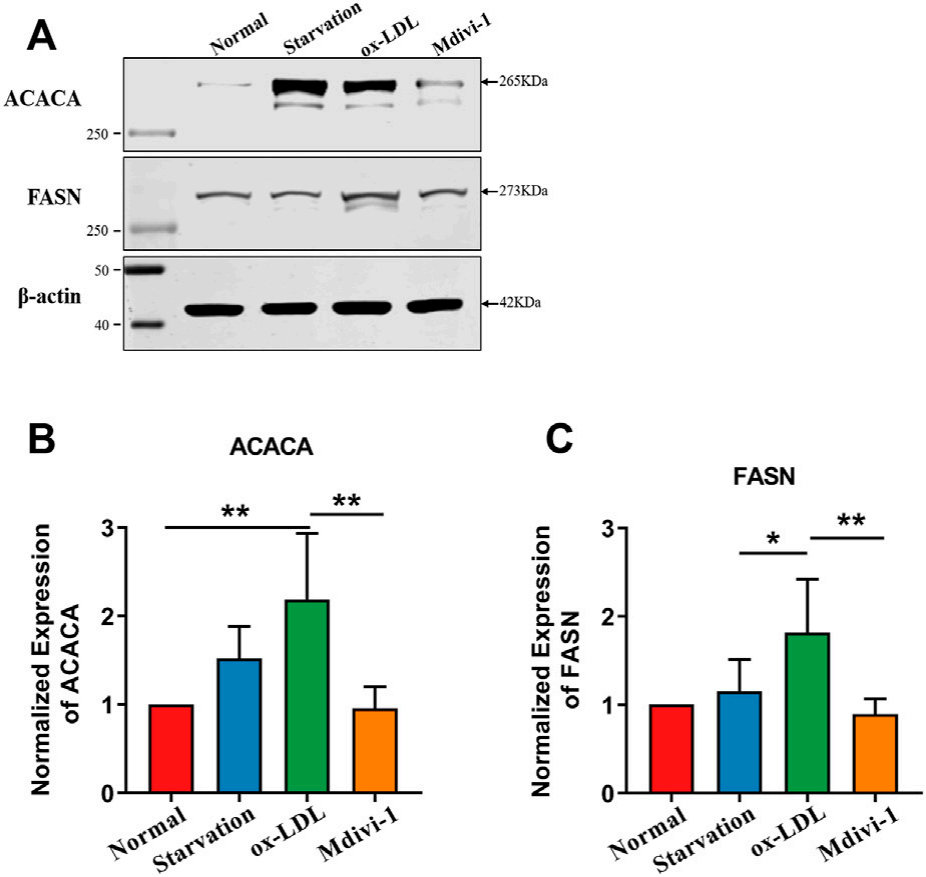
Verification of FASN, ACACA after Mdivi-1 treatment in VSMC.**(A–C)**: Effects of Mdivi-1 on ox-LDL induced the changes of FASN, ACACA expression with western blot. *p < 0.05, **p < 0.01.

In order to explore the mechanism that Mdivi-1 modulated the lipid metabolism of VSMC, we performed the proteomics analysis for ox-LDL and midivi-1 treated VSMC. The results showed that there were 125 differential expressed proteins between ox-LDL group and Mdivi-1 (Figure 6). Among them, Acetyl -CoA carboxylase (ACACA) and fatty acid synthase (FASN), the key regulation enzymes, changed with ox-LDL and Mdivi-1 treated VSMCs, the expression of these two enzymes were identified by western blot, the results showed that ox-LDL treatment significantly increased their expression, Mdivi-1 antagonized the effect of mdivi-1 (Figure 7).

## Discussion

Present study found that mitochondrial fission played important role in VSMC lipid deposition and foaming. Inhibition of mitochondrial fission with Mdivi-1 could effectively antagonize ox-LDL induced VSMC lipid deposition and foaming via improving mitochondrial function and metabolic reprogramming. The mechanism was related with that mdivi-1 inhibited the expression of the key lipid metabolism enzyme, Acetyl -CoA carboxylase (ACACA) and fatty acid synthase (FASN) and then alleviated the synthesis of lipid droplets. The present results provided a new measure for treatment of VSMC lipid deposition in AS.

Previous studies showed that the mechanisms of VSMC foaming mainly involved in the imbalance between lipids in and out of cells (Cheon and Cho, 2021). Ox-LDL entered the cells via LDL receptor (LDLR), and LDL was internalized and transported to the endosomes (Huang et al., 2010). The acidic environment of endosomes altered the conformation of LDLR, promoted the release of LDL and transferred it into the lysosomes for degradation. Several mechanisms participated in the entry of lipids disorder. For example, sterol regulatory element binding protein (SREBP) regulated the transcription level of LDLR, and p38MAPK inhibited the expression of LDLR through p42/44MAPK (Lammi et al., 2014; Lu et al., 2020). In addition to exogenous pathways, the deposition of intracellular lipids could be induced through endogenous pathways. Glycolysis produced pyruvate which entered into mitochondria and catalyzed acetyl CoA by pyruvate dehydrogenase. Acetyl CoA and oxaloacetic acid produced citric acid under the action of citrate synthase. Then, part of citric acid underwent into the tricarboxylic acid cycle (TCA), and the other part of citric acid was back into the cytoplasm through the mitochondrial membrane, which was catalyzed by ATP citrate lyase to produce acetyl CoA and oxaloacetic acid. Acetyl CoA in cytoplasm was an important precursor for fatty acid synthesis (Abo Alrob and Lopaschuk, 2014; Satoh et al., 2020). Acetyl CoA carboxylase (ACACA) catalyzed part of acetyl CoA to produce malonyl COA (Hunkeler et al., 2018). Subsequently, one acetyl CoA molecule and seven malonyl COA molecules were continuously condensed to produce palmitic acid under the action of fatty acid synthase (FASN) (Jiang et al., 2019). Palmitic acid is a saturated fatty acid with 16 carbon atoms which can continue to carry out elongation and desaturation reactions to produce fatty acid molecules with different lengths and saturations. This is also the main reason for intracellular lipid deposition (Korbecki and Bajdak-Rusinek, 2019). Present study found that mitochondrial fission inhibitors could reduce the synthesis of lipids to play a protective effect on VSMC foaming via metabolism reprograming by inhibition of mitochondrial fission, improving mitochondrial function.

Present study demonstrated that ox-LDL stimulation could induce mitochondrial over-fission and dysfunction. Previous studies showed that hyperlipidemia resulted in mitochondrial dysfunction. Previous studies showed that VSMCs presented phenotype transformation from contractile phenotype to synthetic phenotype during the development of AS (Hall et al., 2005). And the number of mitochondria in contractile phenotype of VSMCs was less, while the number of mitochondria in synthetic phenotype of VSMCs was significantly increased. The content of mitochondrial respiratory chain complex I was significantly increased and the coupling between oxidation and phosphorylation was significantly decreased during the dedifferentiation of VSMCs (Formosa et al., 2018). Mitochondrial oxidative phosphorylation of VSMCs in AS plaque was decreased and the glycolysis was increased. There are fewer studies involving hyperlipidemia resulting in mitochondrial excessive fission. While previous study showed that AMPK up-regulated glycolysis of VSMCs by haematopoietic expressed homeobox (hex2), resulting in metabolic reprogramming of VSMCs, which induced the phenotypic transformation of VSMCs and participated in the occurrence of AS (He et al., 2021). With metabonomics and proteomics techniques, we found ox-LDL significantly increased the lipid metabolites of VSMC such as heptadecanoic acid, palmitoleic acid and myristic acid, at same time increased the expression of acetyl-CoA carboxylase (ACACA) and fatty acid synthase (FASN), the key regulation enzyme, resulting in the lipid deposition and VSMC foaming. But how ox-LDL and lipids induce the mitochondrial fission need to be further investigated in the next step.

Mdivi-1 is a classic mitochondrial fission inhibitor by inhibiting the activity of GTPase dynamic related protein (Drp1). Drp1 activation plays a critical role in mitochondrial fission. The phosphorylation of several sites of Drp1 such as Ser 616 and Ser 637 may activate Drp1 GTPase and induces mitochondrial fission (Duan et al., 2020a). So, inhibition of Drp1 GTPase activity is the key measure to suppress mitochondrial fission. Our previous study demonstrated that Mdivi-1 could inhibit the translocation of Drp1 to mitochondria and inhibited mitochondrial fission (Duan et al., 2021). However, several studies found that Mdivi-1 involved in other functions except for inhibiting mitochondrial fission. For example, Duan et al. found that Mdivi-1 inhibited mitochondrial respiratory chain complex I and reduced the generation of ROS in the case of Drp1 deficiency (Duan et al., 2020b). In addition, other inhibitors of mitochondrial fission, P110, had been developed which may improve mitochondrial function and reduce oxidative stress damage. Whether the other effects of Mdivi-1 except for inhibition of Drp1 participate in the inhibitory effect on VSMC foaming needs further investigation.

There were some limitations in present study: the first limitation was that we only investigated in role of ox-LDL in mitochondrial fission and lipid deposition of VSMCs in the present study, but did not investigate the mechanism how ox-LDL induces the mitochondrial fission. The second limitation was that the present study was just performed in cell level, did not confirm the role of Midivi-1 in animal level, this needs to be further verified in next step.

In summary, mitochondrial fission plays an important role in VSMC lipid deposition and foaming, which can be antagonized by inhibition of mitochondrial fission to improve mitochondrial function and metabolic reprogramming. This finding provides a new insight for prevention and treatment of AS.

## Data Availability

The datasets presented in this study can be found in online repositories. The data has been submitted to ProteomeXchange via the PRIDE database with Project accession: PXD035671.
